# Rising incidence of psychiatric disorders before diagnosis of immune-mediated inflammatory disease

**DOI:** 10.1017/S2045796017000579

**Published:** 2017-11-03

**Authors:** R. A. Marrie, R. Walld, J. M. Bolton, J. Sareen, J. R. Walker, S. B. Patten, A. Singer, L. M. Lix, C. A. Hitchon, R. El-Gabalawy, A. Katz, J. D. Fisk, C. N. Bernstein, for the CIHR Team in Defining the Burden and Managing the Effects of Psychiatric Comorbidity in Chronic Immunoinflammatory Disease

**Affiliations:** 1 Department of Internal Medicine, Max Rady College of Medicine, Rady Faculty of Health Sciences, University of Manitoba, Winnipeg, Canada; 2 Department of Community Health Sciences, Max Rady College of Medicine, Rady Faculty of Health Sciences, University of Manitoba, Winnipeg, Canada; 3 Manitoba Centre for Health Policy, Max Rady College of Medicine, Rady Faculty of Health Sciences, University of Manitoba, Winnipeg, Canada; 4 Department of Psychiatry, Max Rady College of Medicine, Rady Faculty of Health Sciences, University of Manitoba, Winnipeg, Canada; 5 Department of Clinical Health Psychology, Max Rady College of Medicine, Rady Faculty of Health Sciences, University of Manitoba, Winnipeg, Canada; 6 Department of Community Health Sciences, Cumming School of Medicine, University of Calgary, Calgary, Canada; 7 Department of Family Medicine, Max Rady College of Medicine, Rady Faculty of Health Sciences, University of Manitoba, Winnipeg, Canada; 8 Department of Anesthesia and Perioperative Medicine, Max Rady College of Medicine, Rady Faculty of Health Sciences, University of Manitoba, Winnipeg, Canada; 9 Departments of Psychiatry, Psychology & Neuroscience, and Medicine, Dalhousie University, Halifax, Canada

**Keywords:** Cohort, epidemiology, inflammatory disease, psychiatry

## Abstract

**Aims.:**

After the diagnosis of immune-mediated inflammatory diseases (IMID) such as inflammatory bowel disease (IBD), multiple sclerosis (MS) and rheumatoid arthritis (RA), the incidence of psychiatric comorbidity is increased relative to the general population. We aimed to determine whether the incidence of psychiatric disorders is increased in the 5 years before the diagnosis of IMID as compared with the general population.

**Methods.:**

Using population-based administrative health data from the Canadian province of Manitoba, we identified all persons with incident IBD, MS and RA between 1989 and 2012, and cohorts from the general population matched 5 : 1 on year of birth, sex and region to each disease cohort. We identified members of these groups with at least 5 years of residency before and after the IMID diagnosis date. We applied validated algorithms for depression, anxiety disorders, bipolar disorder, schizophrenia, and any psychiatric disorder to determine the annual incidence of these conditions in the 5-year periods before and after the diagnosis year.

**Results.:**

We identified 12 141 incident cases of IMID (3766 IBD, 2190 MS, 6350 RA) and 65 424 matched individuals. As early as 5 years before diagnosis, the incidence of depression [incidence rate ratio (IRR) 1.54; 95% CI 1.30–1.84) and anxiety disorders (IRR 1.30; 95% CI 1.12–1.51) were elevated in the IMID cohort as compared with the matched cohort. Similar results were obtained for each of the IBD, MS and RA cohorts. The incidence of bipolar disorder was elevated beginning 3 years before IMID diagnosis (IRR 1.63; 95% CI 1.10–2.40).

**Conclusion.:**

The incidence of psychiatric comorbidity is elevated in the IMID population as compared with a matched population as early as 5 years before diagnosis. Future studies should elucidate whether this reflects shared risk factors for psychiatric disorders and IMID, a shared final common inflammatory pathway or other aetiology.

## Introduction

Various chronic conditions are characterised by immune dysregulation and inflammation that results in acute exacerbations and often progressive disability. Among these immune-mediated inflammatory diseases (IMID), inflammatory bowel disease (IBD), multiple sclerosis (MS) and rheumatoid arthritis (RA) share several epidemiologic and clinical features (Pugliatti *et al.* 2002; Health Canada, 2003; Alamanos *et al.* 2006; Canadian Institute for Health Information, 2007; Logan & Bowlus, 2010; Bernstein *et al.* 2012). As compared with the general population, the incidence and prevalence of psychiatric comorbidity, including depression, anxiety, bipolar disorder and schizophrenia, are increased in individuals with these IMID (Hauser *et al.* 2011; Matcham *et al.* 2013; Marrie *et al.* 2015*b*). This psychiatric comorbidity negatively affects outcomes; depression is associated with lower health-related quality of life in IBD, MS and RA (Rupp *et al.* 2004; Mitchell *et al.* 2005; Faust *et al.* 2012; Mok *et al.* 2012), and with increased mortality in MS and RA (Ang *et al.* 2005; Marrie *et al.* 2015*a*).

Prior studies suggest that the incidence of depression and anxiety may increase before the diagnosis of IMID. A Danish study of 5084 persons with MS and 24 771 age- and sex-matched controls reported increased odds of a diagnosis of depression or anxiety in the 2 years before MS diagnosis but did not evaluate other psychiatric disorders (Hoang *et al.* 2016). In a study of 351 individuals with prevalent IBD that found an increased risk for mood and anxiety disorders, the onset of these psychiatric comorbidities most often pre-dated the diagnosis of IBD (Walker *et al.* 2008). However, this study lacked a matched cohort from the general population for comparison.

The findings in IBD and MS suggest that psychiatric comorbidity may be the earliest manifestation of the onset of IMID in some individuals, and that common underlying aetiologies of psychiatric comorbidity may exist across IMID. Using population-based incident cohorts of three IBD, MS and RA, and matched cohorts from the general population, we aimed to estimate the incidence of several psychiatric disorders, including depression, anxiety, bipolar disorder and schizophrenia, in the 5-year periods pre- and post-IMID diagnosis. We hypothesised that the incidence of psychiatric comorbidity would be higher in the incident IMID than in the matched general population cohorts pre- and post-IMID diagnosis.

## Methods

### Setting

This retrospective cohort study was conducted in the province of Manitoba, Canada. Health care in Canada is a provincial responsibility, and is publicly funded for all residents. Records of health service delivery are prospectively captured electronically and are accessible for research. The University of Manitoba Health Research Ethics Board approved the study, and the Manitoba Health Information Privacy Committee approved data access.

### Data sources

Since 1984, all Manitoba residents have a unique personal health identification number linked to health system contacts. We accessed de-identified administrative databases at the Manitoba Population Research Data Repository of the Manitoba Centre for Health Policy, including the population registry, hospital discharge abstract database (DAD), physician claims and Drug Program Information Network (DPIN). The population registry captures demographic information, dates of health care coverage and residence location as defined by postal code. The DAD captures all hospitalisations including admission and discharge dates, and up to 25 diagnoses. Until 2004, diagnoses were recorded using the International Classification of Disease Ninth revision, clinical modification (ICD)-9-CM codes, and have been recorded using the ICD Tenth revision, Canadian version (ICD-10-CA) thereafter. Physician claims capture the date of service and one diagnosis recorded using ICD-9-CM codes. The DPIN database, which dates from 1995, captures all outpatient prescription dispensations including the date, drug name and drug identification number, which is connected to the World Health Organisation's Anatomical Therapeutic Chemical (ATC) Classification System (WHO Collaborating Centre for Drug Statistics Methodology, 2012). We linked these databases using scrambled, unique provincial identifiers to ensure confidentiality.

### Study populations

First, we applied validated case definitions (Bernstein *et al.* 1999; Marrie *et al.* 2010; Hitchon *et al.* 2014) to the administrative dataset to identify all Manitobans aged 18 years and older with IBD, MS and RA from 1984 to 2013 (Supplementary Table S1). For each condition, we assigned the date of diagnosis (i.e., index date) as the date of the first health claim for that condition. For each condition, we selected a cohort from the general population, matched 5 : 1 on sex, year of birth within ±5 years and region as defined by forward sortation area (first three digits of postal code). These matched cohorts excluded individuals with any diagnostic codes for IBD, demyelinating disease, RA or related disorders as well as anyone with prescriptions for MS-specific disease-modifying therapies (which contributed to the MS case definition) from 1984 to 2013.

Second, we selected incident cases of IBD, MS and RA (and their matched controls) by requiring a 5-year run-in period before the index date without any related claims. A small number of individuals met the case definitions for more than one IMID (*n* = 165). We did not exclude them to ensure generalisability of our findings, but allowed them to count only once in analyses in which we created a combined IMID cohort. In this situation, they were classified on the basis of the IMID with the earliest index date in the coverage period. Third, we limited the analysis to incident cases and controls with at least 5.5 continuous years of data before and after the index date (spanning 11 years). Cases had index dates ranging from 1989 to 2008. We defined the 6-month periods on either side of the index date as the ‘diagnosis year’, the 5 years before the diagnosis year as the pre-diagnosis period and the 5 years after diagnosis as the post-diagnosis period.

### Psychiatric disorders

Using case definitions developed and validated in MS and IBD populations (Marrie *et al.* 2013; Marrie *et al.* 2016), we identified individuals in each cohort with depression, anxiety disorder, bipolar disorder, schizophrenia and any psychiatric disorder (⩾1 of depression, anxiety disorder, bipolar disorder, schizophrenia) (Supplementary Table S1). For each psychiatric disorder, we assigned the diagnosis date as the date of first health claim for that disorder.

### Covariates

Covariates included age at index date [grouped as 18–24 (reference), 25–44, 45–64, ⩾65 years], sex [female *v.* male (reference)], index year [1989–1998 (reference) *v.* 1999–2007], annual number of physician visits unrelated to psychiatric disorders, urban *v.* rural region of residence (urban regions included Winnipeg, population >600 000 and Brandon, population >47 000), and socioeconomic status (SES) at the index year in quintiles (highest quintile of SES as reference). For SES we used the Socioeconomic Factor Index version 2 (SEFI-2), derived by linking postal code to census data. The SEFI-2 combines information regarding average household income, per cent of single-parent households, unemployment rates and high school education rates; scores less than zero indicate better SES (Chateau *et al.* 2012). We also included the number of physician visits to account for possible surveillance bias due to increased health system contacts.

### Analyses

Within each cohort (IBD, MS, RA and their controls), we estimated the incidence of each psychiatric disorder during the diagnosis year, and each year within the pre-diagnosis and post-diagnosis periods. We age- and sex-standardised incidence rates to the 2010 Canadian population, and compared them for each year separately using incidence rate ratios (IRR) and 95% confidence intervals (95% CI) using a negative binomial distribution. We tested whether there were temporal trends in the incidence of psychiatric disorders within the pre- and post-diagnosis periods and whether these trends differed between the pre- and post-diagnosis periods. Findings between the IMID cohorts and matched controls were compared. We constructed multivariable negative binomial regression models that included main effects for case (case *v.* matches), period [pre-diagnosis (reference), diagnosis year, post-diagnosis] and year as a continuous variable (1, 2, 3, 4, 5 in each of the pre-diagnosis and post-diagnosis periods; 0 was the year of diagnosis), as well as two-way interaction terms between case and period, and between year and period, and a three-way interaction between case, year and period. These analyses were conducted in two ways: (ii) combining all IMID into a single ‘case’ group, and all matches into a single ‘matched’ group; and (iii) including a separate variable for each IMID and its matches in the same analysis. These models adjusted for all pre-specified covariates. Given preliminary findings of an elevated incidence of psychiatric disorders as early as 5 years pre-diagnosis, we repeated the analysis, limiting it to incident cases and matches with at least 10.5 continuous years of data before and after the index date (spanning 21 years) to see if the findings were present as early as 10 years pre-diagnosis.

## Results

### Study populations

We identified 12 141 incident IMID cases; 3766 had IBD, 2190 had MS and 6350 had RA. The combined matched controls included 65 424 individuals. In the combined IMID cohort, most were females, living in urban settings at the index date (Table 1). Those in the MS and RA cohorts were more likely to be female than those in the IBD cohort. Those in the RA cohort had a later mean age at diagnosis than those in the IBD or MS cohorts.

**Table 1.** Characteristics of incident disease and matched cohorts at the index date

Characteristic	IBD matches (*n* = 20 355)	IBD (*n* = 3766)	MS matches (*n* = 12 315)	MS (*n* = 2190)	RA matches (*n* = 33 584)	RA (*n* = 6350)	IMID^a^ (*n* = 12 141)	IMID matches (*n* = 65 424)
Female, *n* (%)	11 230 (55.2)	2062 (54.8)	9108 (74.0)	1621 (74.0)	24 692 (73.5)	4658 (73.3)	8220 (67.7)	44 427 (67.9)
Age at diagnosis, mean (s.d.)	41.0 (15.9)	41.5 (16.4)	40.5 (11.7)	40.2 (11.5)	52.3 (15.3)	52.7 (15.4)	47.0 (16.2)	46.6 (16.0)
Duration of follow-up from the index date (years), mean (s.d.)	13.6 (5.1)	13.8 (5.1)	13.7 (5.1)	13.8 (5.0)	13.0 (5.0)	13.0 (4.9)	13.4 (5.0)	13.4 (5.0)
Region of residence, *n* (%)								
Urban	13 545 (66.5)	2491 (66.1)	8172 (66.3)	1435 (65.5)	19 637 (58.5)	3681 (58.0)	7495 (61.7)	40 809 (62.4)
Rural	6810 (33.4)	1275 (33.8)	4143 (33.6)	755 (34.5)	13 947 (41.5)	2669 (42.0)	4646 (38.3)	24 615 (37.6)
Socioeconomic Factor Index score, mean (s.d.)	−0.20 (0.89)	−0.28 (0.92)	−0.20 (0.87)	−0.27 (0.90)	0.08 (1.00)	0.04 (1.00)	−0.11 (0.99)	−0.06 (0.96)

IBD, inflammatory bowel disease; MS, multiple sclerosis; RA, rheumatoid arthritis; IMID, immune-mediated inflammatory disease, combining IBD, MS and RA cohorts; socioeconomic status, Socioeconomic Factor Index scores; values less than zero indicate higher socioeconomic status.

a A small number of individuals met the case definitions for more than one of the IMIDs of interest. We did not exclude them to ensure generalisability of our findings (since IMID can be comorbid with each other), but only allowed them to count once in the IMID cohort. In this case, they were classified on the basis of the IMID with the earliest index date in the coverage period.

### Incidence of psychiatric disorders in the combined IMID cohort

**Fig. 1.**
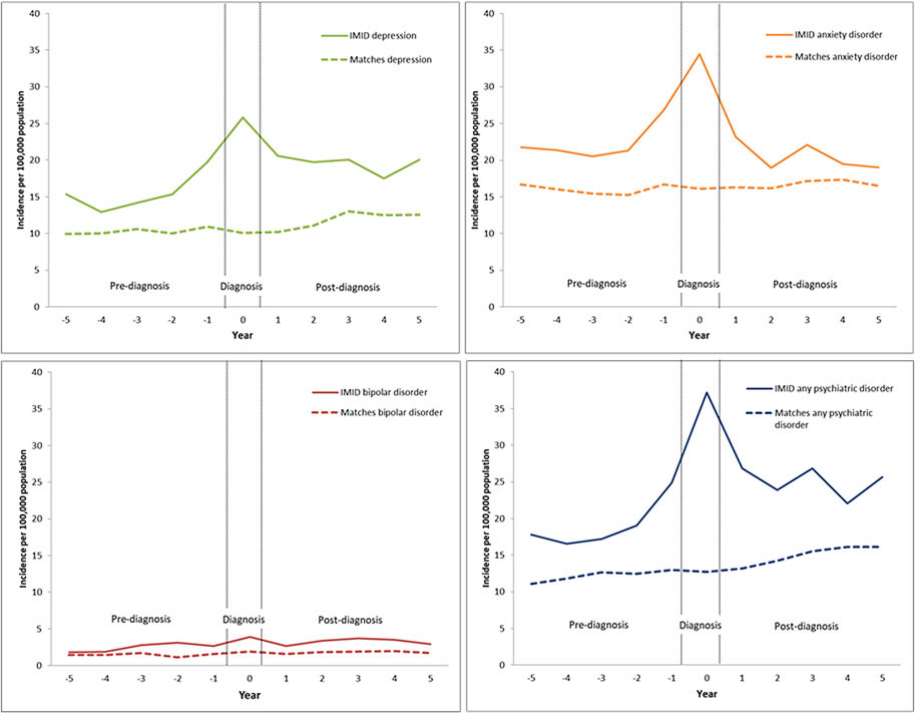
Age- and sex-standardised incidence rates of psychiatric disorders in the immune-mediated inflammatory disease (IMID) and matched cohorts 5 years before and 5 years after IMID diagnosis.

In the combined IMID cohort, the age- and sex-standardised incidence of any psychiatric disorder rose during the pre-diagnosis period, peaked during the diagnosis year, then declined slightly post-diagnosis but remained higher than in the pre-diagnosis period (Fig. 1).

**Fig. 2.**
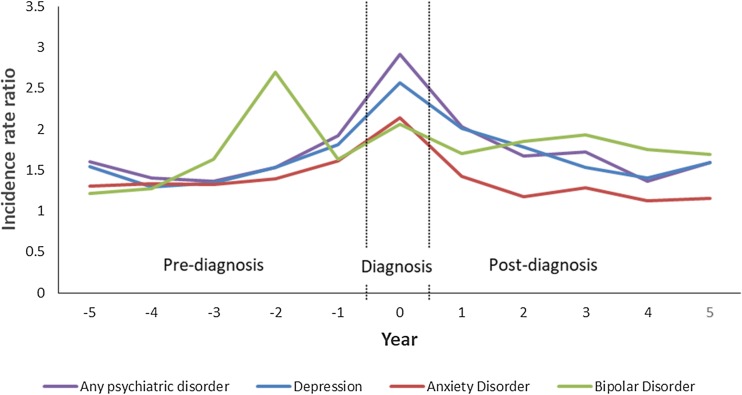
Age- and sex-standardised incidence rate ratios for psychiatric disorders in the immune-mediated inflammatory disease (IMID) and matched cohorts 5 years before and 5 years after IMID diagnosis.

The age- and sex-standardised incidence of any psychiatric disorder among the IMID cohort was higher than the matched cohort during all periods, beginning as early as 5 years before IMID diagnosis (IRR 1.60; 95% CI 1.36–1.89), and most prominently in the diagnosis year (Fig. 2, Table 2).

**Table 2.** Incidence rate ratios (95% confidence intervals) comparing age- and sex-standardised incidence of psychiatric disorders in the year of diagnosis^a^

	IBD	MS	RA	IMID
Any psychiatric disorder	3.11 (2.44–3.95)	4.66 (3.03–7.17)	2.52 (1.85–4.34)	2.92 (2.56–3.32)
Depression	2.76 (2.10–3.62)	3.62 (2.49–5.27)	2.25 (1.57–3.23)	2.57 (2.21–2.99)
Anxiety disorder	2.50 (1.97–3.18)	2.90 (2.13–3.96)	1.42 (1.07–1.89)	2.14 (1.87–2.44)
Bipolar disorder	1.73 (0.93–3.23)	3.29 (1.78–6.08)	1.38 (0.82–2.33)	1.88 (1.35–2.62)
Schizophrenia	^b^	^b^	^b^	1.71^c^ (0.73–4.01)

a Age- and sex-standardised to the 2010 Canadian population.

b Suppressed due to small numbers.

c Crude rate ratio as number of those affected too few to standardise incidence rates.

Similarly, the adjusted regression analyses showed that in the IMID cohort, the incidence of any psychiatric disorder rose in the pre-diagnosis period, and declined slightly during the post-diagnosis period (Table 3). In the matched cohort, the incidence of any psychiatric disorder rose slightly over time, but this pattern was the same in the pre- and post-diagnosis periods.

**Table 3.** Adjusted incidence rate ratios (95% confidence intervals) for the association between immune-mediated inflammatory disease (IMID) and psychiatric disorders

Variable	Any psychiatric disorder	Depression	Anxiety disorder	Bipolar disorder
Combined IMID cohort				
Year: cases pre-index	1.08 (1.03–1.13)	1.07 (1.02–1.12)	1.03 (0.98–1.07)	1.11 (0.99–1.24)
Year: cases post-index	0.93 (0.90–0.96)	0.95 (0.92–0.98)	0.89 (0.86–0.92)	0.98 (0.91–1.05)
Post-pre ratio: cases	0.86 (0.81–0.90)	0.89 (0.84–0.94)	0.87 (0.82–0.92)	0.88 (0.77–1.00)
Year: controls pre-index	1.03 (1.01–1.04)	1.01 (0.99–1.03)	1.00 (0.98–1.01)	1.04 (1.00–1.08)
Year: controls post-index	1.04 (1.03–1.06)	1.04 (1.02–1.06)	1.01 (0.98–1.03)	1.01 (0.97–1.05)
Post-pre ratio: controls	1.02 (0.99–1.05)	1.03 (1.00–1.07)	1.02 (0.99–1.04)	0.98 (0.91–1.05)

The year variable assesses whether there is an annual linear increase in incidence in the group (cases or controls) and period (pre-index or post-index) of interest. Post-pre ratio compares the year effect in the post-index *v.* pre-index periods. A ratio less than one indicates the yearly rise in incidence was greater in the pre-index than the post-index period. A ratio greater than one indicates the yearly rise in incidence was greater in the post-index than the pre-index period.

When we examined the incidence of depression, the findings were similar to those for any psychiatric disorder (Fig. 1, Tables 2 and 3). For anxiety disorders, the pattern of the findings was similar to those for any psychiatric disorder and depression (Fig. 1). Although the incidence of anxiety disorder was higher in the IMID cohort than in the matched cohort, this was not statistically significant in the fourth and fifth years post-diagnosis (IRR 1.12; 95% CI 0.94–1.34; IRR 1.15; 95% CI 0.96–1.38). While the incidence of anxiety was higher in the IMID cohort than in the matched cohort pre-diagnosis, the regression analyses did not demonstrate any change in the incidence of anxiety over the pre-diagnosis period (Table 3).

For bipolar disorder, we observed a similar pattern of findings, including an increased incidence in the IMID cohort relative to the matched cohort emerging 3 years before IMID diagnosis (IRR 1.63; 95% CI 1.10–2.40) (Fig. 1, Table 2). Although the incidence of bipolar disorder rose during the pre-diagnosis period, this temporal change did not reach statistical significance (Table 3).

The incidence of schizophrenia in the IMID cohort was not statistically significantly higher in the diagnosis year than in the matched cohort either in the diagnosis year (Table 2), or pre-diagnosis. However, these findings should be viewed cautiously given the small number of individuals affected in each cohort; the small numbers also precluded regression analyses.

### Incidence of psychiatric disorders in the individual IMID cohorts

In the analyses of each IMID cohorts (IBD, MS and RA), the findings were generally consistent in magnitude and direction with those of the combined IMID cohort (Supplementary Table S2 and Figs S1–S3). In the diagnosis year, the age- and sex-standardised incidence of any psychiatric disorder, depression and anxiety disorder was higher in each IMID cohort as compared with its matched cohort (Table 2). In the IBD cohort, the incidence of any psychiatric disorder and depression were elevated relative to its matched cohort beginning 5 years pre-diagnosis, and rose over the pre-diagnosis period. The incidence of anxiety disorder was significantly elevated beginning 3 years pre-diagnosis. The incidence of any psychiatric disorder, depression and anxiety disorder were elevated beginning 5 years pre-diagnosis in the MS cohort *v.* its matched cohorts and rose over the pre-diagnosis period. In RA, the incidence of anxiety disorder was elevated as compared with the matched cohort at all times, but did not rise significantly over the pre-diagnosis period. In the IBD, MS and RA cohorts, the incidence of bipolar disorder was elevated as compared with the matched cohorts beginning to 2–3 years pre-diagnosis, but the rise in incidence over the pre-diagnosis period did not reach statistical significance (Supplementary Fig. S3). The data for schizophrenia could not be modelled due to small cell sizes.

### Sensitivity analyses

When we restricted the analyses to members of the combined IMID and matched cohorts with 10 years of data before and after the diagnosis year, characteristics of the cohorts were similar to those of the original cohort (Supplementary Table S3), as were the findings in the diagnosis year (Supplementary Table S4). The pattern of findings regarding the incidence of the psychiatric disorders was also similar in that the increased incidence of any psychiatric disorder was already evident 10 years pre-diagnosis (IRR 1.50; 95% CI 1.10–2.06) in the combined IMID cohort (Supplementary Fig. S4), and 8–10 years pre-diagnosis in the individual IMID cohorts (data not shown).

## Discussion

Using population-based data and an observational design, we evaluated whether the incidence of psychiatric disorders increases before the initial diagnosis of IMID. Unlike previous studies, we studied three IMID simultaneously to identify common patterns, and we evaluated multiple psychiatric disorders. We focused on incidence rather than prevalence because this is more relevant for understanding the aetiology of disease, and provides greater clarity in understanding of the temporal associations between IMID and psychiatric disorders. As compared with age-, sex- and geographically matched cohorts, the incidence of psychiatric comorbidity was increased in the IMID cohorts in the 5–10 years before IMID diagnosis. The similarity in findings across three IMID suggests a common underlying biology for psychiatric disorders in IMID.

Our findings are consistent with a limited number of prior studies that have suggested higher than expected incidence or prevalence of depression and anxiety before the diagnosis of IBD or MS (Kurina *et al.* 2001; Walker *et al.* 2008; Byatt *et al.* 2011; Hoang *et al.* 2016); we were unable to identify comparable studies for bipolar disorder. In one study of 187 individuals with IBD and a lifetime history of any mood or anxiety disorder, 18% had onset of these disorders within 2 years preceding IBD diagnosis and an additional 16% had onset 2–9 years before diagnosis (Walker *et al.* 2008). A study of the ulcerative colitis form of IBD that used physician claims data from one health region in Alberta, Canada found that neuroses/depression were more likely to occur before IBD diagnosis although psychotic disorders did not. A British case–control study using administrative data found that the prevalence of depression and anxiety was higher than expected in the year before diagnosis of ulcerative colitis, and the prevalence of depression was higher more than 5 years before diagnosis (Kurina *et al.* 2001). A Danish study using administrative data found that the odds of a diagnosis of depression or anxiety were higher in MS *v.* matched controls in the 2-year period before MS diagnosis (OR 1.4; 95% CI 1.05–1.88) (Hoang *et al.* 2016). In an interview-based study of 29 individuals with MS, 17 had a lifetime prevalence of major depressive disorder, of whom four reported this as a prodrome preceding neurologic symptom onset (Byatt *et al.* 2011).

We found that the incidence of depression and anxiety disorder was increased substantially in the year of IMID diagnosis. The period surrounding the diagnosis of chronic illness can be associated with increased depressive symptoms that may or may not worsen over time (Chiu *et al.* 2016). One British study suggested that the risk of depression and anxiety were highest in the first year after diagnosis of IBD (Kurina *et al.* 2001). In MS, nearly half of newly diagnosed individuals report high levels of anxiety or depressive symptoms (Janssens *et al.* 2003). In RA, over one-third of individuals report high levels of emotional distress at diagnosis (Persson *et al.* 2005), and 80% of depressive disorders are diagnosed within 5 years (Wang *et al.* 2014). In a large Danish study using health claims data, several autoimmune diseases were associated with an increased risk of bipolar disorder within the first 4 years after diagnosis (thyrotoxicosis, MS, IBD, psoriasis, RA), or 5 or more years after diagnosis (autoimmune hepatitis) (Eaton *et al.* 2010). Similarly, several autoimmune diseases were associated with an increased risk of schizophrenia within the first 4 years after diagnosis (thyrotoxicosis, iridocyclitis, psoriasis, Sjogren's syndrome) or 5 or more years after diagnosis (thyrotoxicosis, Crohn's disease, autoimmune hepatitis). Collectively, this suggests the risk of developing psychiatric disorders is particularly high in the first 4 or 5 years post-diagnosis.

Several possibilities may explain the increased incidence of psychiatric disorders during the pre-diagnosis period for IMID. First, this could reflect a prodromal period for the IMID in which inflammation has developed sufficiently to increase the risk of psychiatric disorders but not to precipitate typical clinical manifestations of IMID. Emerging evidence suggests that the risk of depression, anxiety disorders and bipolar disorder is influenced by the inflammation and activation of cell-mediated immunity (Vieira *et al.* 2010; Berk *et al.* 2013; Rosenblat & McIntyre, 2015). Second, psychiatric disorders and IMID may share common aetiologic factors. Some genetic loci are associated with the risk of both psychiatric and immune-mediated disorders (Wang *et al.* 2015). As well, environmental factors such as social adversity and chronic stress may induce biological changes which subsequently increase the risk of one or more chronic diseases (Cohen *et al.* 2012). In autoimmune disease, for example, common aetiologic factors with differing local pathogenetic effects may manifest as chronic immune disorders in one or more organ systems. Third, there may be a surveillance bias in the IMID cohorts, in which individuals begin to present to the health system for non-specific manifestations of IMID such as fatigue, thereby increasing their likelihood of obtaining diagnoses of other conditions. For example, a study of 5305 Americans with newly diagnosed MS found that 28.9% had seen a physician for fatigue, as based on billing codes, during the 3-year period before MS diagnosis; comparative data in individuals without MS were not available (Berger *et al.* 2013). Nonetheless, our point estimates were unchanged after adjusting for the annual number of physician visits. Fourth, the onset of psychiatric disorders could increase the subsequent risk of developing IMID. Depression is known to induce changes in immune function, including immunosuppression and inflammation (Nair *et al.* 1999; Yeragani *et al.* 2000; Irwin & Miller, 2007), as well as hyperactivity of the hypothalamic pituitary axis (Stetler & Miller, 2011). Individuals with generalised anxiety disorder may also have dysregulated T-cell function (Vieira *et al.* 2010). In addition, psychiatric disorders are associated with adverse health behaviours, while serious psychiatric disorders are associated with inequalities in health care provision that may lead to other health conditions (Lawrence & Kisely, 2010). All of these aforementioned explanations largely consider the occurrence of psychiatric disorders in persons with IMID to be distinct comorbid conditions. However, the occurrence of psychiatric disorders pre-diagnosis of IMID could potentially be conceptualised as early symptoms of IMID rather than as distinct comorbid conditions. While this possibility would have important implications for the diagnostic evaluation and treatment of individuals with incident psychiatric disorders, our study does not allow us to distinguish these possibilities. Moreover, it is likely that psychiatric disorders are comorbid conditions in some persons with IMID but direct effects of IMID in other persons with IMID. Future studies are necessary to evaluate all of the potential explanations for the association between psychiatric disorders and subsequent development of IMID. Such studies would benefit from measuring genetic factors, as well as environmental factors including infectious exposures, health behaviours and measures of social adversity in longitudinal population-based cohorts with and without psychiatric disorders. Further pathobiological study of mental health diseases in the context of IMID using brain imaging and serological and tissue biomarker studies will also be needed to discern the pathogenesis of psychiatric disorders in IMID (Najjar *et al.* 2013; Pearlman & Najjar, 2014)

This study was limited to a single Canadian province potentially reducing generalisability. However, we have found in previous studies of MS that our findings regarding the relative risk of psychiatric comorbidity after MS diagnosis and temporal trends are consistent across other Canadian provinces (Marrie *et al.* 2015*b*). Administrative data are not collected for research, but we have validated our approaches to identifying IBD, MS, RA and psychiatric comorbidity using these data (Bernstein *et al.* 1999; Marrie *et al.* 2010; Marrie *et al.* 2013; Marrie *et al.* 2016). These data only capture individuals who have sought care through hospitals or physician visits and do not capture individuals with psychiatric disorders diagnosed by non-physician mental health providers, nor those who have not sought any treatment, although this would affect individuals with and without IMID. As administrative data lack clinical details, we could not evaluate associations between the timing of onset of psychiatric comorbidity and specific characteristics of the IMID, such as severity of illness.

We found that the incidence of psychiatric comorbidity is increased as early as 5–10 years before the diagnosis of IMID. Future studies should elucidate whether this reflects shared risk factors for psychiatric disorders and IMID, a shared final common inflammatory pathway or other aetiology. From a clinical perspective, the increased risk of psychiatric comorbidity at the time of IMID diagnosis and post-diagnosis highlights the clinical importance of detecting and treating these comorbidities in an appropriate, timely fashion. Detection will require clinical awareness and efficient screening strategies, while treatment will require effective collaborative mental health models. Our findings also raise questions regarding appropriate diagnostic investigations when individuals without an apparent history of IMID present with incident psychiatric disorders; future studies will need to address this issue.

## Supplementary material

For supplementary material accompanying this paper visit https://doi.org/10.1017/S2045796017000579. click here to view supplementary material

## Availability of Data and Materials

The authors received permission to access the data used in this study; however, they are unable to share the data as they are not the data custodians.
