# Supplementary material for: Rising incidence of psychiatric disorders before diagnosis of immune-mediated inflammatory disease
Source: Epidemiol Psychiatr Sci. 2017 Nov 3;28(3):333–42. doi: 10.1017/S2045796017000579 (PMC6998907; doi:10.1017/S2045796017000579)

**eTable 1. Case definitions for identifying immune-mediated inflammatory diseases and psychiatric comorbidity in administrative health data**

| **Condition** | **ICD-9-CM/ICD-10-CA** | **ATC** | **No. Years of Data*** | **No. & Type of Claims** |
| --- | --- | --- | --- | --- |
| Inflammatory bowel disease[^19^](#_ENREF_19) | 555.xx, 556.xx/K50, K51 | - | <2 | ≥3 (H or P) |
|  |  |  | >2 | ≥5 (H or P) |
| Multiple sclerosis[^20^](#_ENREF_20) | 340/ G35 | L03AX13, L03AB07, L03AB08, N07XX09, L04AA23, L04AA27, L04AA31, L04AA34 | All years available | ≥3 (H or P or Rx) |
| Rheumatoid arthritis[^21^](#_ENREF_21) | 714.0/ M05, M06 | - | <2 | ≥5 (H or P) |
|  |  |  | >2 | ≥3 (H or P or Rx) |
| Depression^b^ | 296.2, 296.3, 298.0, 300.4, 311/ F32, F33, F34 | N06AA01, N06AA02, N06AA04, N06AA11, N06AA12, N06AA17, N06AA21, N06AB03, N06AB04, N06AB05, N06AB06, N06AB08, N06AB10, N06AF03, N06AF04, N06AG02, N06AX06, N06AX11, N06AX16, N06AX21, N06AX23, | 2 | ≥1 H or ≥5 P OR (≥1 P AND ≥7 Rx) |
| Anxiety^c^ |  | N05AB12 (alprazolam), N05AB06 (lorazepam) | 2 | ≥1 H or ≥2 P OR (≥1 P AND ≥2 Rx) |
| Bipolar disorder[^22^](#_ENREF_22) | 296.0, 296.1, 296.04, 296.14, 296.4, 296.44, 296.5, 296.44, 296.5, 296.54, 296.6, 296.7, 296.8/ F31 | - | 5 | ≥1 H or ≥3 P |
| Schizophrenia[^22^](#_ENREF_22) | 295.x/ F20.x, F25 | - | 2 | ≥1 H or ≥2 P |

ICD = International Classification of Disease, ATC = Anatomic Therapeutic Chemical Classification System, H = hospital claims; P = physician claims, Rx = prescription claims

*Length of period (contiguous) over which claims must be accumulated. For IBD and RA definitions varied depending on number of years of data available

a-Anticonvulsants used were restricted to lamotrigine, carbamazepine, and valproate which are used as mood stabilizers, after excluding persons with epilepsy. b-Antidepressants excluded amitriptyline, nortriptyline and buspirone because of their frequent off-label uses. c-None of the antidepressants were considered specific for anxiety. Most benzodiazepines were also considered not to be adequately specific for anxiety disorders.

eTable 2. Incidence rate ratios (95% confidence intervals) for the association between immune-mediated inflammatory disease (IMID) and psychiatric disorders

| **Variable** | **Any psychiatric disorder** | | **Depression** | | **Anxiety disorder** | | **Bipolar disorder** | |
| --- | --- | --- | --- | --- | --- | --- | --- | --- |
|  | **Unadjusted** | **Adjusted** | **Unadjusted** | **Adjusted** | **Unadjusted** | **Adjusted** | **Unadjusted** | **Adjusted** |
| *Inflammatory bowel disease* |  |  |  |  |  |  |  |  |
| Year: Cases pre-index | 1.05  (0.97, 1.14) | 1.05  (0.97, 1.14) | 1.02  (0.93, 1.11) | 1.02  (0.93, 1.11) | **1.05**  **(0.97, 1.14)** | 1.05  (0.97, 1.13) | 1.12  (0.93, 1.36) | 1.13  (0.94, 1.36) |
| Year: Cases post-index | **0.89**  **(0.84, 0.94)** | **0.89**  **(0.84, 0.95)** | **0.89**  **(0.84, 0.95)** | **0.89**  **(0.84, 0.95)** | **0.88**  **(0.83, 0.94)** | **0.88**  **(0.83, 0.94)** | 0.99  (0.88, 1.13) | 0.99  (0.88, 1.13) |
| Post-pre ratio: Cases | **0.85**  **(0.76, 0.94)** | **0.85**  **(0.77, 0.94)** | **0.88**  **(0.79, 0.98)** | **0.88**  **(0.79, 0.98)** | **0.84**  **(0.76, 0.93)** | **0.84**  **(0.76, 0.93)** | 0.88  (0.70, 1.11) | 0.88  (0.70, 1.10) |
| Year: Controls pre-index | **1.05**  **(1.00, 1.10)** | **1.04**  **(1.01, 1.07)** | 1.04  (0.99, 1.10) | 1.02  (0.99, 1.06) | **0.99**  **(0.95, 1.04)** | 1.01  (0.98, 1.03) | 1.09  (0.97, 1.23) | 1.08  (1.00, 1.16) |
| Year: Controls post-index | **1.06**  **(1.02, 1.10)** | **1.06**  **(1.03, 1.09)** | **1.05**  **(1.02, 1.10)** | **1.05**  **(1.02, 1.08)** | **1.01**  **(0.98, 1.04)** | 1.02  (0.99, 1.04) | 1.03  (0.95, 1.11) | 1.03  (0.96, 1.10) |
| Post-pre ratio: Controls | 1.01  (0.95, 1.07) | 1.02  (0.97, 1.07) | 1.02  (0.95, 1.08) | 1.03  (0.97, 1.09) | 1.02  (0.96, 1.07) | **1.01**  **(0.97, 1.06)** | 0.94  (0.72, 1.22) | 0.95  (0.82, 1.08) |
|  |  |  |  |  |  |  |  |  |
| *Multiple sclerosis* |  |  |  |  |  |  |  |  |
| Year: Cases pre-index | **1.18**  **(1.08, 1.29)** | **1.18**  **(1.08, 1.29)** | **1.18**  **(1.07, 1.30)** | **1.18**  **(1.07, 1.30)** | **1.09**  **(1.00, 1.20)** | **1.09**  **(1.00, 1.20)** | 1.16  (0.92, 1.45) | 1.16  (0.92, 1.45) |
| Year: Cases post-index | **0.82**  **(0.77, 0.87)** | **0.82**  **(0.77, 0.87)** | **0.85**  **(0.80, 0.91)** | **0.85**  **(0.80, 0.91)** | **0.79**  **(0.74, 0.86)** | **0.79**  **(0.74, 0.85)** | 0.89  (0.76, 1.04) | 0.89  (0.76, 1.04) |
| Post-pre ratio: Cases | **0.69**  **(0.62, 0.77)** | **0.69**  **(0.62, 0.77)** | **0.72**  **(0.64, 0.81)** | **0.72**  **(0.64, 0.81)** | **0.73**  **(0.65, 0.82)** | **0.73**  **(0.65, 0.81)** | 0.77  (0.58, 1.01) | 0.77  (0.58, 1.01) |
| Year: Controls pre-index | **1.07**  **(1.02, 1.13)** | **1.03**  **(1.00, 1.07)** | **1.06**  **(1.00, 1.12)** | 1.02  (0.99, 1.06) | 1.02  (0.97, 1.07) | 1.01  (0.98, 1.04) | 1.05  (0.93, 1.19) | 1.05  (0.96, 1.14) |
| Year: Controls post-index | 1.02  (0.98, 1.07) | 1.01  (0.97, 1.04) | 1.01  (0.97, 1.05) | 0.99  (0.96, 1.03) | 1.00  (0.96, 1.04) | 0.99  (0.93, 1.03) | 0.98  (0.89, 1.07) | 0.98  (0.90, 1.06) |
| Post-pre ratio: Controls | 0.96  (0.89, 1.02) | 0.98  (0.92, 1.03) | 0.95  (0.89, 1.02) | **0.97**  **(0.91, 1.03)** | **0.98**  **(0.92, 1.05)** | **0.99**  **(0.93, 1.05)** | 0.93  (0.79, 1.09) | 0.93  (0.81, 1.08) |
|  |  |  |  |  |  |  |  |  |
| *Rheumatoid arthritis* |  |  |  |  |  |  |  |  |
| Year: Cases pre-index | 1.06  (0.99, 1.13) | 1.06  (0.99, 1.12) | 1.06  (0.99, 1.13) | 1.06  (0.99, 1.13) | 0.99  (0.94, 1.05) | 0.99  (0.94, 1.05) | 1.07  (0.90, 1.26) | 1.07  (0.90, 1.27) |
| Year: Cases post-index | 1.01  (0.97, 1.06) | 1.01  (0.97, 1.06) | 1.05  (0.99, 1.10) | **1.05**  **(1.00, 1.10)** | 0.96  (0.91, 1.01) | 0.96  (0.92, 1.01) | 1.03  (0.92, 1.15) | 1.03  (0.92, 1.15) |
| Post-pre ratio: Cases | 0.96  (0.89, 1.03) | 0.96  (0.89, 1.03) | 0.99  (0.91, 1.08) | 0.99  (0.91, 1.08) | 0.97  (0.89, 1.05) | 0.97  (0.90, 1.04) | 0.96  (0.79, 1.18) | 0.96  (0.79, 1.18) |
| Year: Controls pre-index | 1.02  (0.98, 1.06) | **1.02**  **(1.00, 1.04)** | 1.00  (0.96, 1.04) | 1.00  (0.97, 1.02) | 0.99  (0.95, 1.02) | 0.98  (0.96, 1.00) | 0.97  (0.89, 1.05) | 1.02  (0.97, 1.08) |
| Year: Controls post-index | **1.04**  **(1.02, 1.07)** | **1.04**  **(1.02, 1.07)** | **1.05**  **(1.02, 1.08)** | **1.05**  **(1.02, 1.07)** | 1.01  (0.99, 1.04) | 1.01  (0.99, 1.03) | 0.99  (0.93, 1.05) | 1.01  (0.96, 1.07) |
| Post-pre ratio: Controls | 1.02  (0.98, 1.07) | 1.03  (0.99, 1.07) | **1.05**  **(1.00, 1.10)** | **1.05**  **(1.01, 1.10)** | 1.03  (0.98, 1.07) | 1.03  (0.99, 1.07) | 1.03  (0.92, 1.14) | 0.99  (0.90, 1.09) |

The year variable assesses whether there is an annual linear increase in incidence in the group (cases or controls) and period (pre-index or post-index) of interest. Post-pre ratio compares the year effect in the post-index versus pre-index periods. A ratio less than one indicates the yearly rise in incidence was greater in the pre-index than the post-index period. A ratio greater than one indicates the yearly rise in incidence was greater in the post-index than the pre-index period.

**eTable 3**. Characteristics of incident disease cohorts at the time of diagnosis, and matched cohorts with 10 years of follow-up before and after the index date, at the index date.

| **Characteristic** | **IBD matches**  **(n = 7,684)** | **IBD**  **(n = 1,477)** | **MS matches**  **(n = 4,896)** | **MS**  **(n = 885)** | **RA matches**  **(n = 12,960)** | **RA**  **(n = 2,470)** | **IMID matches**  **(n = 25,263)** | **IMID**  **(n = 4,773)** |
| --- | --- | --- | --- | --- | --- | --- | --- | --- |
| Female, n (%) | 4,287 (55.8) | 828 (56.0) | 3,606 (73.6) | 656 (74.1) | 9,624 (74.2) | 1,841 (74.5) | 17,297 (68.5) | 3,278 (68.7) |
| Age at diagnosis, mean (SD) | 40.4 (15.1) | 40.9 (15.6) | 40.5 (11.1) | 40.0 (10.9) | 51.2 (14.7) | 51.5 (14.7) | 45.8 (15.2) | 46.0 (15.4) |
| Follow-up from the index date (years), mean (SD) | 14.3 (2.2) | 14.3 (2.3) | 14.2 (2.2) | 14.2 (2.2) | 14.1 (2.3) | 14.1 (2.3) | 14.2 (2.3) | 14.2 (2.3) |
| Region of residence, n (%) |  |  |  |  |  |  |  |  |
| Urban | 5,079 (66.1) | 972 (65.8) | 3,217 (65.7) | 585 (66.1) | 7,473 (57.7) | 1,428 (57.8) | 15,580 (61.7) | 2,942 (61.6) |
| Rural | 2,605 (33.9) | 505 (34.2) | 1,679 (34.3) | 300 (33.9) | 5,487 (42.3) | 1,042 (42.2) | 9,683 (38.3) | 1,831 (38.4) |
| Socioeconomic status | -0.19 (0.89) | -0.29 (0.95) | -0.22 (0.86) | -0.31 (0.90) | 0.09 (1.02) | 0.03 (1.07) | -0.05 (0.96) | -0.13 (1.02) |

IBD = inflammatory bowel disease, MS = multiple sclerosis, RA = rheumatoid arthritis, IMID = immune-mediated inflammatory disease, combining IBD, MS and RA cohorts

**eTable 4. Incidence rate ratios (95% confidence intervals) comparing age and sex-standardized incidence of psychiatric disorders in the year of diagnosis^a^** in cohorts with 10 years of follow-up before and after the index date

|  | **IBD** | **MS** | **RA** | **IMID** |
| --- | --- | --- | --- | --- |
| Any psychiatric disorder | 3.40  (2.31, 5.01) | 3.79  (2.32, 6.18) | 3.34  (2.03, 5.49) | 3.10  (2.5, 3.79) |
| Depression | 3.57  (2.31, 5.51) | 3.29  (1.86, 5.83) | 3.12  (1.84, 5.29) | 3.07  (2.44, 3.85) |
| Anxiety disorder | 2.63  (1.40, 4.02) | 2.37  (1.40, 4.02) | 1.36  (0.90, 2.03) | 2.16  (1.74, 2.69) |
| Bipolar disorder | c | 4.23  (1.37, 13.0) | 4.02  (1.10, 14.7) | 2.64  (1.51, 4.62) |
| Schizophrenia | c | c | c | c |

a-Age and sex-standardized to the 2010 Canadian population; b-crude rate ratio as number of those affected too few to standardize incidence rates; c= suppressed due to small numbers

**Figure e1. Age- and sex-standardized incidence rates of psychiatric disorders in the inflammatory bowel disease (IBD) and matched cohorts five years before and five years after index date**


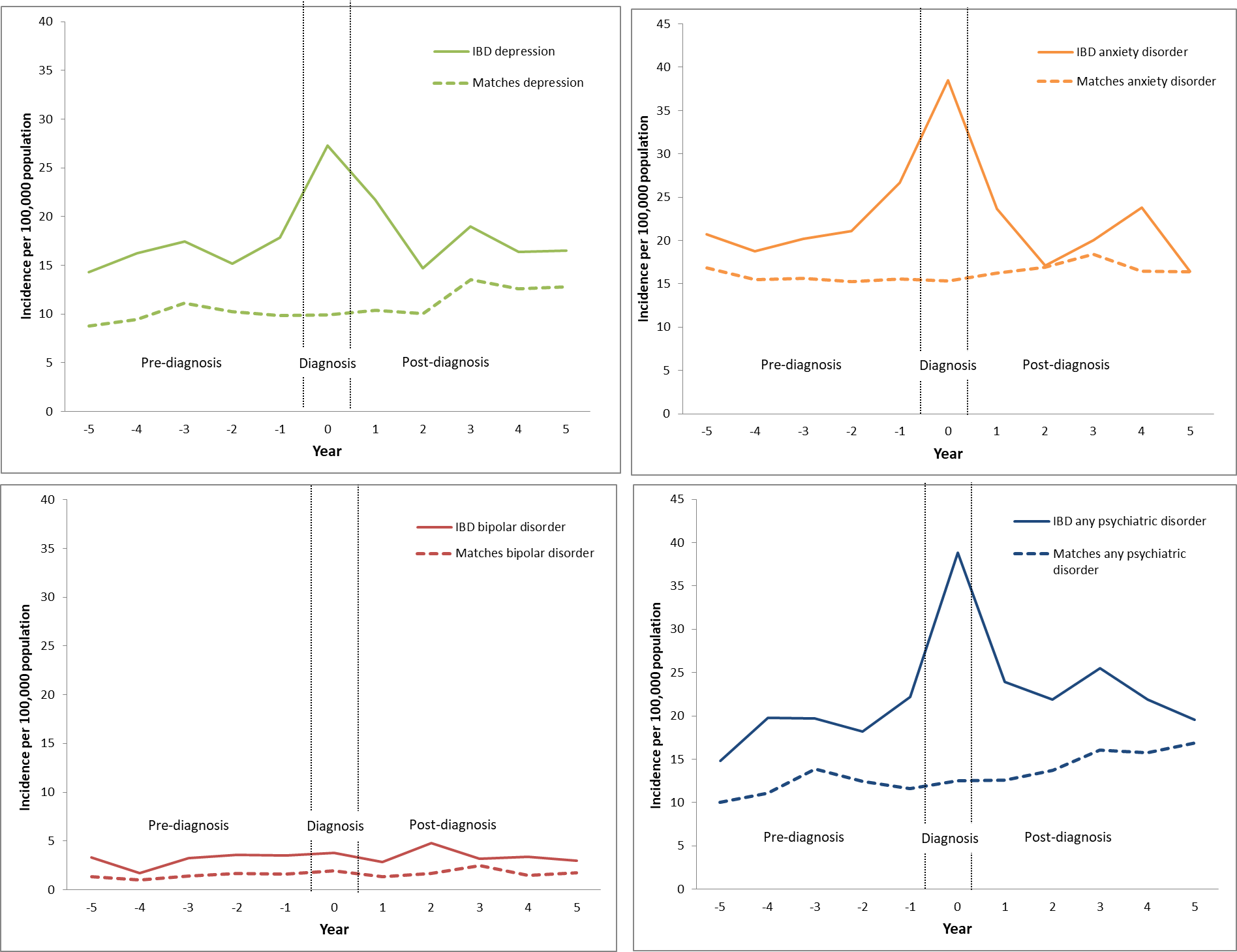


**Figure e2. Age- and sex-standardized incidence rates of psychiatric disorders in the multiple sclerosis (MS) and matched cohorts five years before and five years after index date**


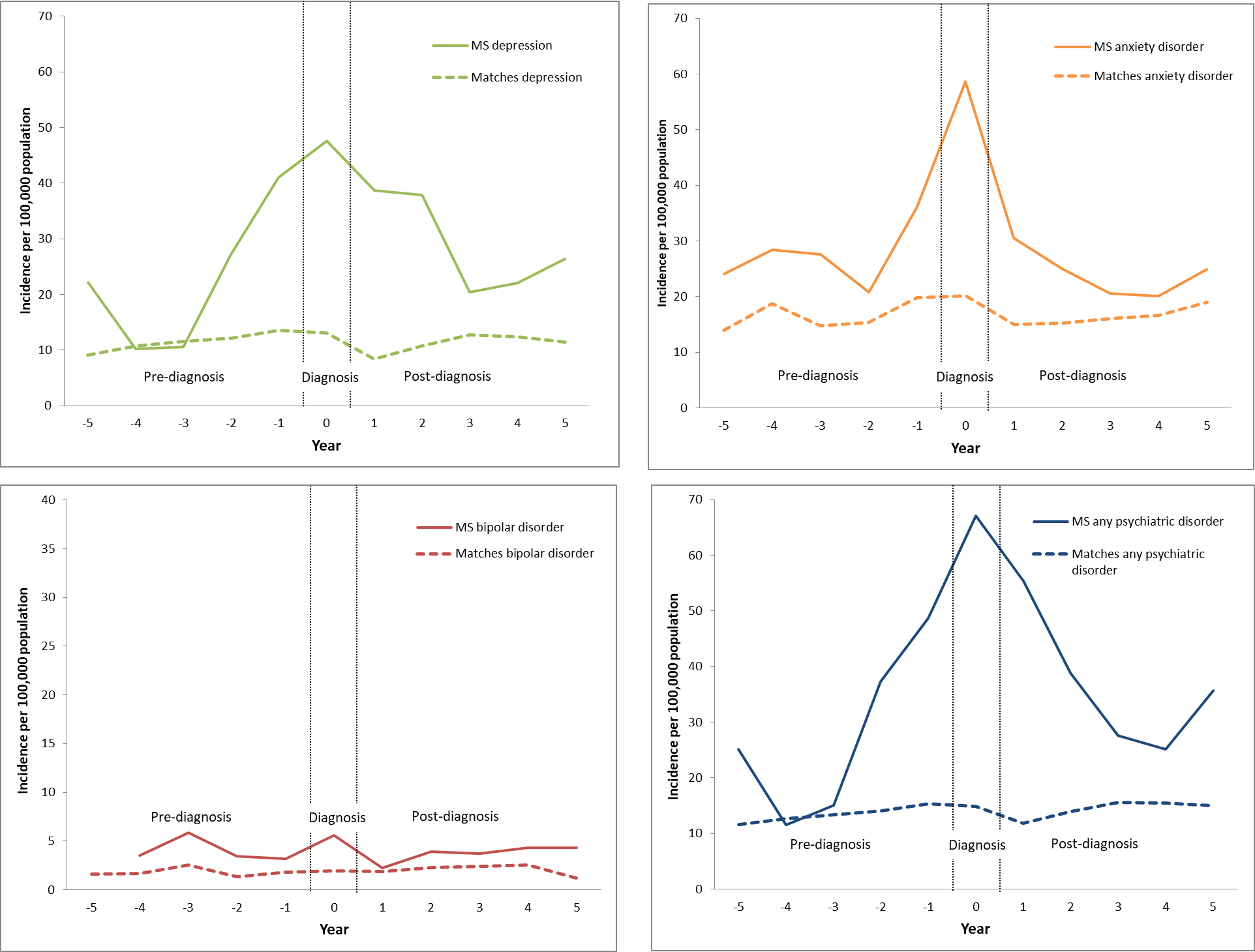


**Figure e3. Age- and sex-standardized incidence rates of psychiatric disorders in the rheumatoid arthritis (RA) and matched cohorts five years before and five years after index date**


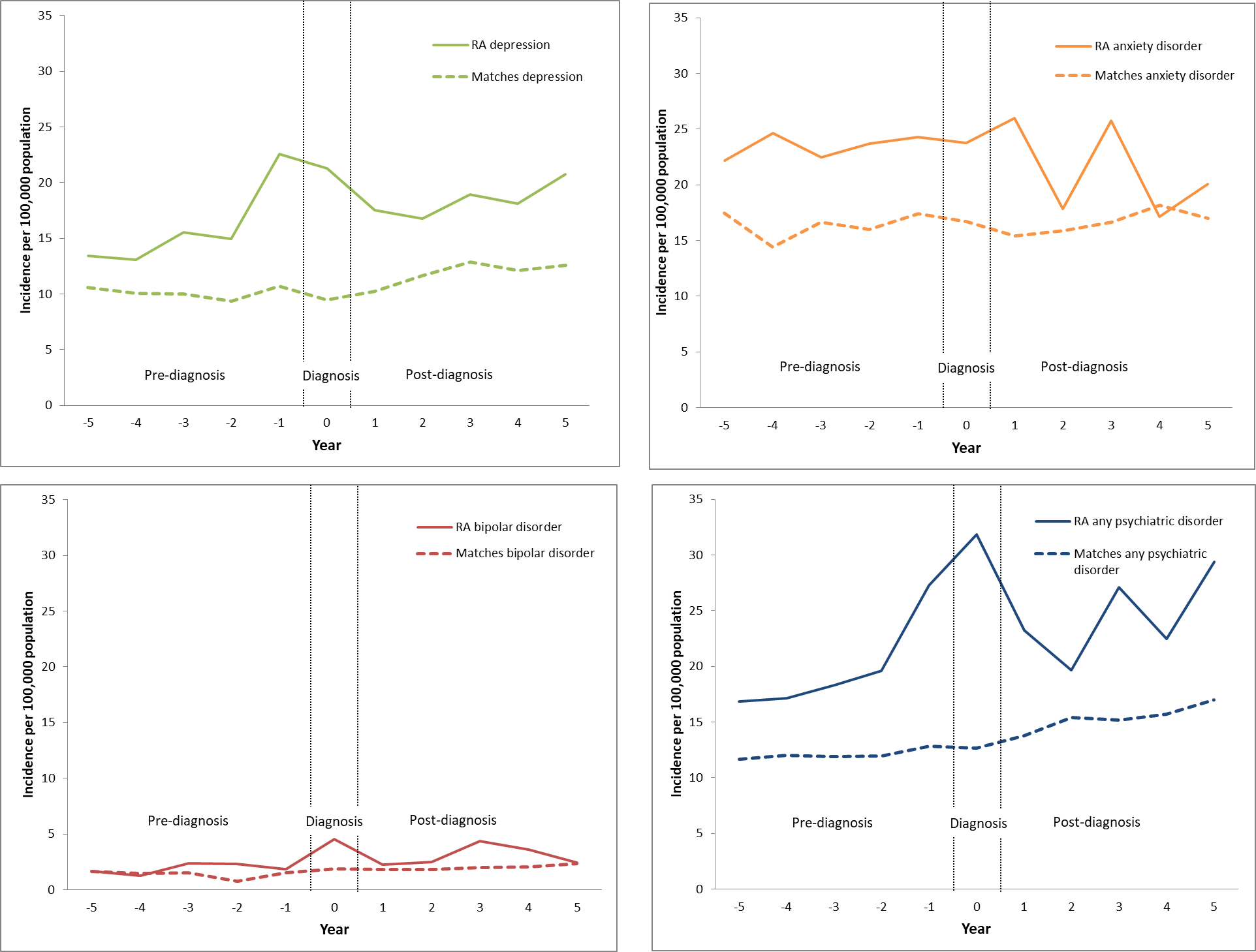


**Figure e4. Age- and sex-standardized incidence rates of psychiatric disorders in the immune-mediated inflammatory disease (IMID) and matched cohorts ten years before and ten years after IMID diagnosis**


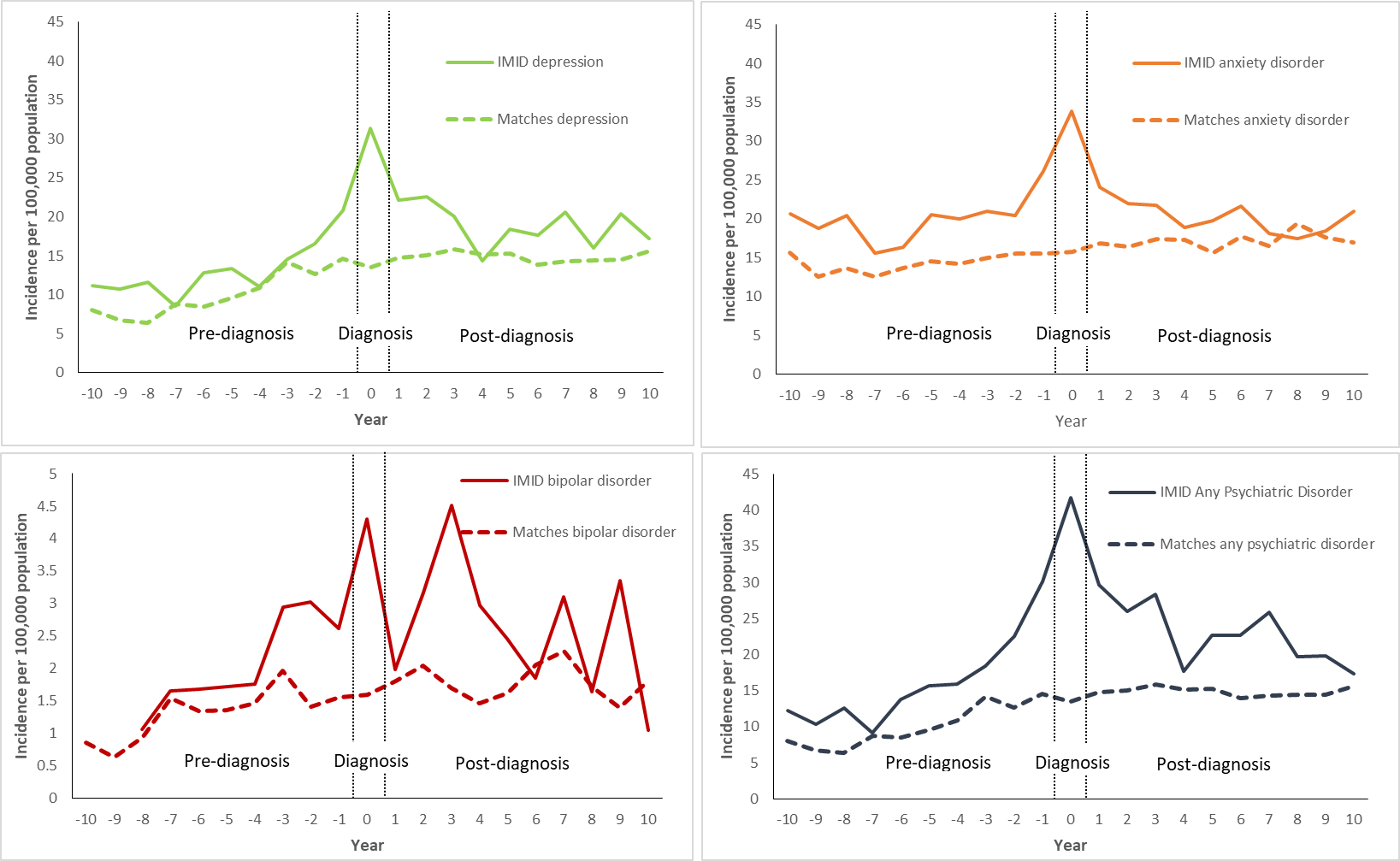

Supplement: Supplementary file 1 [file S2045796017000579sup001.doc]
